# Targeted cell imaging properties of a deep red luminescent iridium(iii) complex conjugated with a c-Myc signal peptide†
†Electronic supplementary information (ESI) available: Experimental synthetic procedures, HPLC data, additional photophysical data and DNA binding data. See DOI: 10.1039/c9sc05568a


**DOI:** 10.1039/c9sc05568a

**Published:** 2020-01-08

**Authors:** Adam H. Day, Martin H. Übler, Hannah L. Best, Emyr Lloyd-Evans, Robert J. Mart, Ian A. Fallis, Rudolf K. Allemann, Eman A. H. Al-Wattar, Nathaniel I. Keymer, Niklaas J. Buurma, Simon J. A. Pope

**Affiliations:** a School of Chemistry , Cardiff University , Main Building , Cardiff , CF10 3AT , UK . Email: popesj@cardiff.ac.uk; b School of Biosciences , Cardiff University , Sir Martin Evans Building , Cardiff , UK

## Abstract

A nuclear localisation sequence (NLS) peptide, PAAKRVKLD, derived from the human c-Myc regulator gene, has been functionalised with a long wavelength (*λ*_ex_ = 550 nm; *λ*_em_ = 677 nm) cyclometalated organometallic iridium(iii) complex to give the conjugate **Ir-CMYC**. Confocal fluorescence microscopy studies on human fibroblast cells imaged after 18–24 h incubation show that **Ir-CMYC** concentrations of 80–100 μM promote good cell uptake and nuclear localisation, which was confirmed though co-localisation studies using Hoechst 33342. In comparison, a structurally related, photophysically analogous iridium(iii) complex lacking the peptide sequence, **Ir-PYR**, showed very different biological behaviour, with no evidence of nuclear, lysosomal or autophagic vesicle localisation and significantly increased toxicity to the cells at concentrations >10 μM that induced mitochondrial dysfunction. Supporting UV-visible and circular dichroism spectroscopic studies show that **Ir-PYR** and **Ir-CMYC** display similarly low affinities for DNA (*ca.* 10^3^ M^–1^), consistent with electrostatic binding. Therefore the translocation and nuclear uptake properties of **Ir-CMYC** are attributed to the presence of the PAAKRVKLD nuclear localisation sequence in this complex.

## Abstract 2

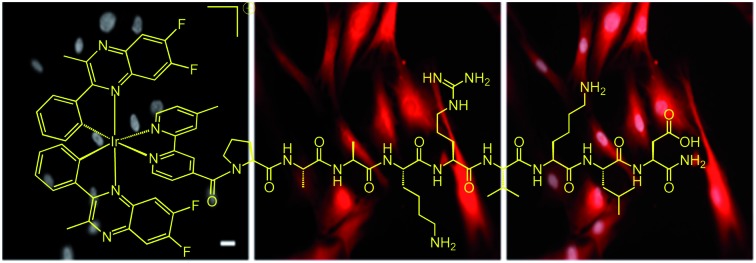
Visualising a c-Myc nuclear localisation signal peptide using an organometallic complex.

## Introduction

A signal peptide is a short peptide sequence often present at the N-terminus of newly synthesised proteins that is responsible for intra- and extracellular translocation.^1^ When that translocation target is the cell nucleus such a peptide can facilitate transport of macromolecules between the cytoplasm and the nucleus and is referred to as a nuclear localisation signal (NLS).^2^ The best characterised mechanisms for protein import across the nuclear envelope are described for the “classical” NLSs, which consists of either one (PKKKRKV) or two (KRPAATKKAGQAKKKK) amino acid sequences.^3^ Previous research has shown that >50% of nuclear proteins conform to these sequences.^4^

We were interested in an alternative NLS peptide sequence (PAAKRVKLD) which is derived from the human c-Myc protein (residues 320–328) and was first identified in 1988 to be essential for nuclear localisation of the protein.^5^ The PAAKRVKLD sequence is somewhat unusual as a NLS because three of its residues are cationic at physiological pH.^5^ The c-Myc protein is the transcription regulator expressed from the c-Myc gene, whose constitutive expression in cancer is associated with increased expression of other genes involved in cell proliferation, contributing to cancer development.^6^ It is tightly regulated in non-cancerous cells, but is now understood to be a frequently deregulated oncogene.^7^ Therefore c-Myc is of interest when considering approaches to targeted cancer therapy.^8^ Fusion proteins of the c-Myc NLS with β-galactosidase have also been used as a positive control for the identification of other nuclear localisation sequences,^9^ and in the production of light-inducible nuclear localisation sequences for tracking protein dynamics in live cells.^10^ Recently, a study described how green fluorescent protein (GFP) loaded nanoparticulate assemblies have been used to deliver proteins directly to the cytosol of cells. Conjugation of GFP with a variety of NLSs enabled observable trafficking of the protein to the nucleus using confocal fluorescence microscopy.^11^ These results showed that the c-Myc NLS conjugate produced the highest relative intensity of fluorescence in the nucleus *versus* the cytosol.

In this study, we wished to visualise the cellular translocalisation characteristics of the c-Myc NLS (PAAKRVKLD) in human fibroblast cells. To do this we investigated the use of a deep red luminescent organometallic complex as an optical label for the c-Myc NLS, which we envisaged would be advantageous when applied to confocal fluorescence microscopy. The potential of luminescent transition metal complexes (*e.g.* based upon Ru(ii), Re(i), Ir(iii) and Pt(ii)) for cellular bioimaging has been explored over the last decade.^12^ In some cases metal complexes functioning as probes for cellular dysfunction have also been investigated.^13^ However, in biological and bioimaging studies it is notoriously difficult to predict the intracellular localisation behaviour of such species because of the challenging interplay of charge, hydro- and lipophilicity, structure–function relationships, and cytotoxicities.^14^ This in turn makes the rational design of organometallic bioimaging probes extremely challenging. The use of targeting vectors is one way to address this challenge.

Only a small number of reports have detailed the use of signal peptide conjugates of luminescent coordination complexes. Examples include rhenium(i) bisquinoline complexes for targeting the folate receptor of cancer cells,^15^ and ^99m^Tc(i) labelled compounds to investigate targeting of radioimaging (and therapeutic) agents.^16^ Polypyridine complexes of Ru(ii) have been the most developed in this area. Keyes and co-workers have reported a cell permeable polyarginine–Ru(ii) complex possessing triplet metal-to-ligand charge transfer (^3^MLCT) emission behaviour and associated oxygen quenching sensitivity.^17^ Subsequent studies by the same group have described multimodal variants,^18^ peptide bridged dinuclear Ru(ii) complexes for monitoring oxygen concentration in cells,^19^ and nuclear targeting with a NLS (VQRKRQKLMP) Ru(ii) conjugate^20^ leading to photoinduced DNA destruction.^21^ Related 1,4,5,8-tetraazaphenanthrene (TAP) ligands on Ru(ii) have also been studied, because of their photooxidising properties that can induce significant DNA damage, although interestingly the phototoxicity can be strongly inhibited by peptide (VQRKRQKLMP) conjugation of the complex.^22^ The cell nucleus is clearly an attractive target when considering the delivery of phototherapeutic action and transition metal complexes, particularly those of the 4^th^ and 5^th^ rows, have long been investigated for applications to photodynamic therapy (PDT).^23^

Herein we present an example of a peptide functionalised luminescent Ir(iii) complex, and, to the best of our knowledge, the first small molecule luminescent moiety to feature a c-Myc inspired NLS. Together with an appropriately designed structural variant that lacks the NLS, this study demonstrates that the c-Myc NLS enables transport of the Ir(iii) conjugate into the nucleus of human fibroblast cells.

## Results and discussion

### Synthesis of the NLS peptide and the Ir(iii) complexes

**Scheme 1:**
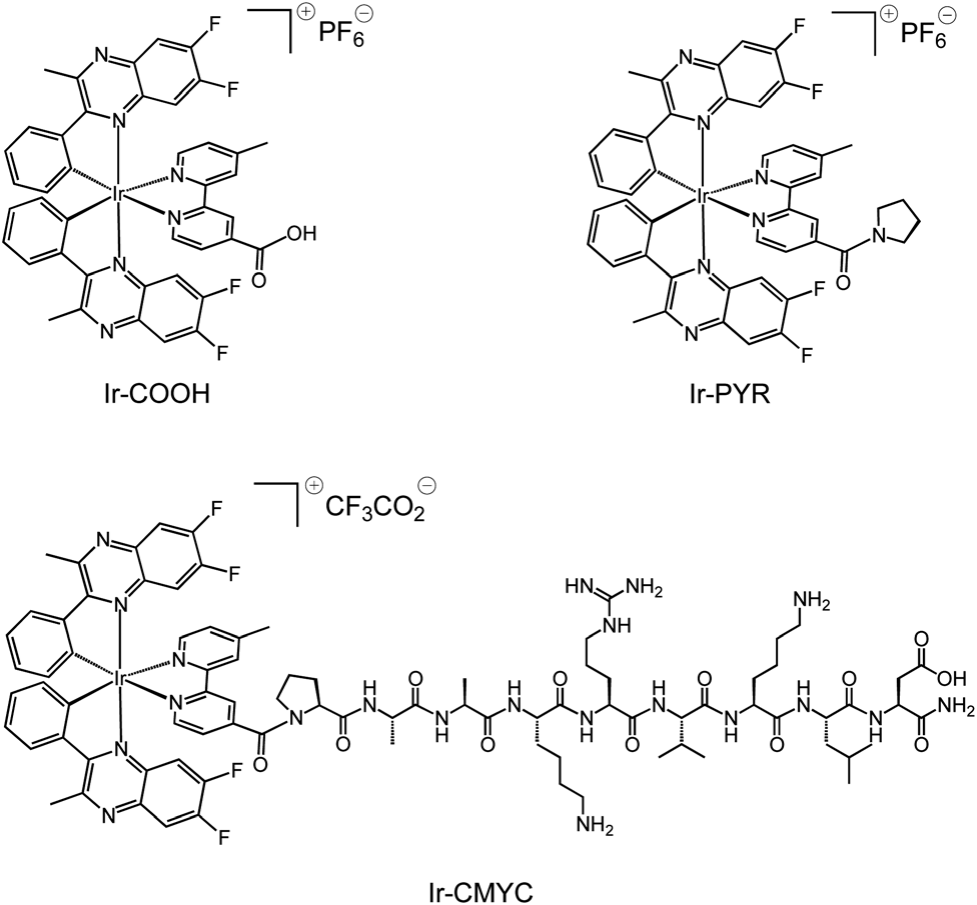
Structures of the luminescent complexes **Ir-COOH**, **Ir-PYR** and **Ir-CMYC**, investigated during this study.

The adopted strategy was to conjugate an organometallic iridium(iii) complex entity to the N-terminus of the peptide *via* an amide bond whilst retaining the intrinsic luminescent properties of the complex. The cyclometalated iridium(iii) complexes (Scheme 1) were synthesised according to previous methods described for related systems.^24^ Thus, the difluorinated quinoxaline ligand (6,7-difluoro-2-methyl-3-phenylquinoxaline) was used as a cyclometalating ligand to form the dimetallic precursor [(L)_2_Ir(μ-Cl)_2_Ir(L)_2_], which was then split using 4-methyl-2,2′-bipyridine-4-carboxylic acid to give monometallic **Ir-COOH**.

For the PAAKRVKLD sequence, Fmoc-solid phase peptide synthesis was performed using the Rink amide resin (0.19 mmol g^–1^ loading) at 0.1 mmol scale using standard techniques. Coupling was performed using 5.0 eq. *O*-(benzotriazol-1-yl)-*N*,*N*,*N*′,*N*′-tetramethyluronium hexafluorophosphate, 5.0 eq. 1-hydroxybenzotriazole, *N*,*N*-diisopropylethylamine and 4.0 eq. of amino acid. Single coupling was performed for 60 min, while double coupling was carried out for 45 min per cycle for arginine and valine and their direct successive amino acids. Subsequent Fmoc deprotection was achieved using standard conditions (see Experimental) to afford the crude peptide attached to the resin. **Ir-COOH** was then coupled in identical conditions to afford the crude iridium peptide complex. The crude iridium–peptide complex was cleaved off the resin by stirring in (v/v/v/v) 92.5 : 2.5 : 2.5 : 2.5 TFA/water/DODT/TIS (where TFA = trifluoroacetic acid, DODT = 2,2′-(ethylenedioxy)diethanethiol, TIS = triisopropylsilane) for 2 hours at room temperature to yield 5 mg of crude conjugate. LC-MS analysis using a linear gradient of 10% to 95% acetonitrile–water (+0.1% TFA) over 13 min revealed the crude **Ir-CMYC** which eluted at 8.0 min, with the molecular composition of the iridium–peptide complex confirmed by LC-MS with observed *m*/*z* = 947 and 632, corresponding to the [M + H]^2+^ and [M + 2H]^3+^ of **Ir-CMYC**, respectively. The crude conjugate was purified *via* preparatory HPLC (Fig. S2 and S3, ESI†) under an identical gradient eluting at 28.6 min, prior to lyophilization yielding 1.5 mg of pure **Ir-CMYC.** The lyophilized **Ir-CMYC** was subsequently used for all characterization and biological testing.

**Fig. 1:**
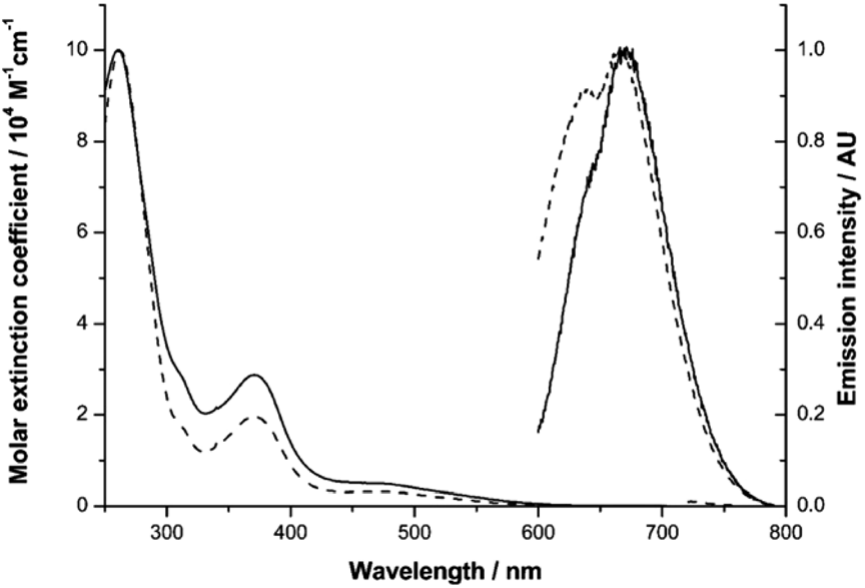
UV-vis absorption and emission spectra of **Ir-PYR** (dashed line) and **Ir-CMYC** (solid line).

An additional iridium(iii) complex lacking the peptide sequence, **Ir-PYR**, was also synthesised for investigation as a photophysical analogue of **Ir-CMYC**. Thus, 4-methyl-2,2′-bipyridine-4-carboxylic acid was simply converted to 4′-methyl-(2,2′-bipyridine)-4-(pyrrolidin-1-yl)methanone to give a new bipyridine derivative (see ESI† for experimental procedure), and then in turn reacted with [(L)_2_Ir(μ-Cl)_2_Ir(L)_2_] to give the pyrrolidine terminated species **Ir-PYR**. **Ir-PYR** was purified using preparatory HPLC (Fig. 1, ESI) and characterised using the standard array of techniques: ^1^H NMR data was particularly insightful, revealing the three unique methyl proton environments (two from the inequivalent quinoxalines, one from the bipyridine), as well as the pyrrolidine protons at 3.2–1.8 ppm. High resolution MS was also obtained confirming the parent cation with the correct isotopic distribution at *m*/*z* = 968.2446 (Fig. S1, ESI†).

### Photophysical characterisation of **Ir-PYR** and **Ir-CMYC**

Our previous studies on Ir(iii) complexes have shown that the use of substituted, cyclometalated quinoxaline ligands can impart red luminescence characteristics.^25^ Firstly, the UV-vis absorption and emission spectral characteristics of **Ir-PYR** were determined. The absorption spectrum was obtained in water and showed a composite of bands between 200–600 nm; the appearance of the spectrum is reminiscent of closely related [Ir(C^N)_2_(N^N)]^+^ complexes of this type.^26^ By inference, ligand-centred transitions are assumed to dominate below 350 nm, with both quinoxaline and bipyridine located π → π* contributing to the spectrum. At 350–450 nm it is likely that spin-allowed metal-to-ligand (5d-to-π*) charge transfers (^1^MLCT) are present, with a weaker (*ε* < 1000 M^–1^ cm^–1^) shoulder feature at 450–600 nm, which is presumably due to a spin forbidden (S_0_ → T_1_) ^3^MLCT absorption. The corresponding absorption spectrum for the peptide functionalised species **Ir-CMYC** shares the same features with **Ir-PYR**, showing that the addition of the peptide sequence does not dramatically alter the intrinsic electronic properties of the complex. To assess longer term stability in water, UV-vis absorption spectra of an aqueous solution (stored at 25 °C in light) of **Ir-CMYC** were recorded at 0, 24, 48 and 72 h (Fig. S4, ESI†). The resultant spectra were superimposable suggesting not only good chemical stability, but also excellent solubility.

The luminescence properties of the Ir(iii) complexes (Fig. 1) were determined in dilute aerated aqueous solvent. Initially, an excitation wavelength of 550 nm was adopted which is selective for the MLCT absorption bands of the complex, and resulted in a broad emission peak (*λ*_em_) for **Ir-PYR** centred at 674 nm (*cf.***Ir-COOH***λ*_em_ = 673 nm). The long wavelength emission characteristics are consistent with our previous studies on cyclometalated quinoxaline [Ir(C^N)_2_(N^N)]^+^ complexes of Ir(iii).^25^ The modest bathochromic shift is likely induced by the polar nature of the water solvent, which in turn suggests a ^3^MLCT contribution to the emitting state. For **Ir-PYR** time-resolved measurements gave the corresponding lifetime (*τ*_obs_) at 42 ns, and a quantum yield assessment gave *ca.* 3%, which is typical for this class of compound in aerated solvent. The phosphorescent nature of **Ir-PYR** was demonstrated by a recorded lifetime of 353 ns in MeCN (Fig. S5, ESI†). Luminescence spectroscopy on **Ir-CMYC** gave comparable data (Fig. 1), with *λ*_em_ = 677 nm (*τ*_obs_ = 38 ns), thus confirming that the photophysical properties of the Ir(iii) complex moiety are retained after functionalisation with the peptide sequence.

### Cell imaging with **Ir-PYR** and **Ir-CMYC**

**Fig. 2:**
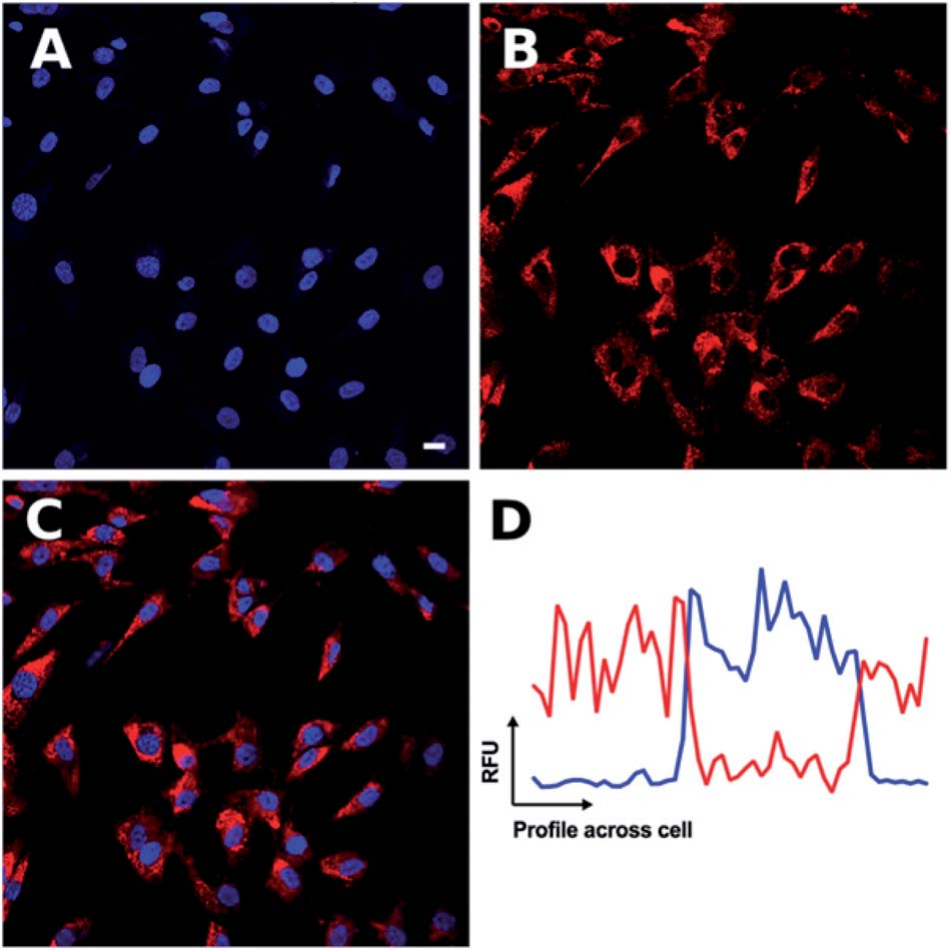
Confocal microscopy shows **Ir-PYR** to produce cytoplasmic punctate staining. **Ir-PYR** (red, (B)) was incubated with human fibroblasts at a concentration of 5 μM for 18 hours, and the nuclei with Hoechst 33342 (blue, (A)). Merged channels (C) demonstrate the lack of **Ir-PYR** nuclear co-localization, as represented in a sample co-localization plot (D) showing the relative fluorescent units (RFU) of both **Ir-PYR** (red) and Hoechst 33342 (blue) across a cell. Representative scale bar in (A) is equal to 7.5 μm.

The non-peptide functionalised complex **Ir-PYR** was investigated at a range of concentrations (1, 5, 20, 50, 80, 100 μM) with respect to cell uptake, localization and bioimaging capability. Human fibroblast cells were incubated with the different concentration solutions of **Ir-PYR** and imaged after 18 hours using confocal fluorescence microscopy. With long wavelength excitation and emission parameters (*λ*_ex_ = 480 nm; *λ*_em_ = 645 nm) compatible with the spectral properties of the complex, it was clearly evident (Fig. 2) that the deep red emission of **Ir-PYR** was highly amenable to confocal fluorescence microscopy.

**Fig. 3:**
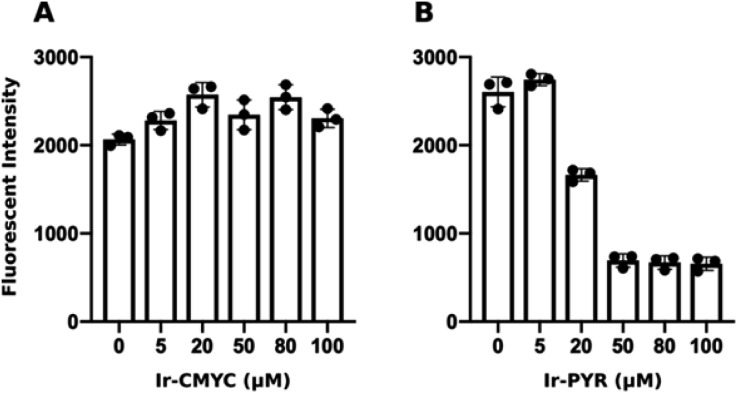
**Ir-PYR** is cytotoxic at 20 μM and above. Human fibroblasts were incubated with various concentrations (0 or DMSO only, 5, 20, 50, 80, 100 μM) of either **Ir-CMYC** (A) or **Ir-PYR** (B) for 18 hours. Resazurin was added to cells, and after a 4 hour incubation the relative fluorescence intensity was measured (*λ*_ex_ = 530 nm; *λ*_em_ = 580 nm). **Ir-PYR** treated cells (>20 μM) show reduced conversion of resazurin to the fluorescent resorufin indicating reduced cell viability. No cytotoxicity was observed at any **Ir-CMYC** concentration.

**Fig. 4:**
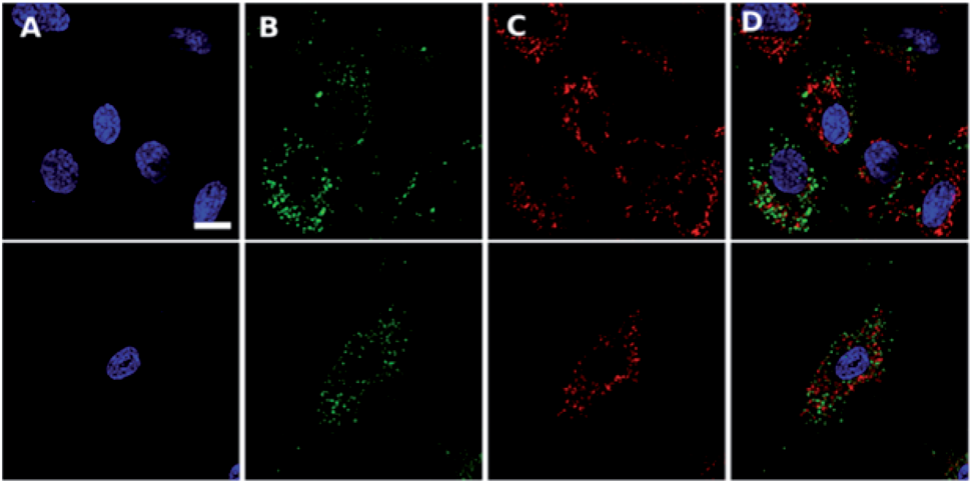
Confocal scanning microscopy shows **Ir-PYR** does not co-localize with nuclei, lysosomes or autophagic vesicles. **Ir-PYR** (red, (C)) was incubated with human fibroblasts at 5 μM for 18 hours and co-stained with Hoechst 33342 (blue, (A)) and either LysoTracker green (top row, (B)) or CYTO-ID (second row, (B)). Merged channels (D) demonstrate the lack of **Ir-PYR** co-localization with any utilised counterstain. Representative scale bar in (A) is equal to 7.5 μm.

At lower concentrations **Ir-PYR** showed punctate staining of the cells, but with no evidence for nuclear localization (Fig. 2), whereas at 50–100 μM the compound appeared to be very toxic, as evidenced by reduced cell number and mitochondrial dysfunction (Fig. 3).^27^ A series of co-localization imaging experiments revealed that **Ir-PYR** does not localise in the nucleus, lysosomes or autophagic vesicles (Fig. 4).

**Fig. 5:**
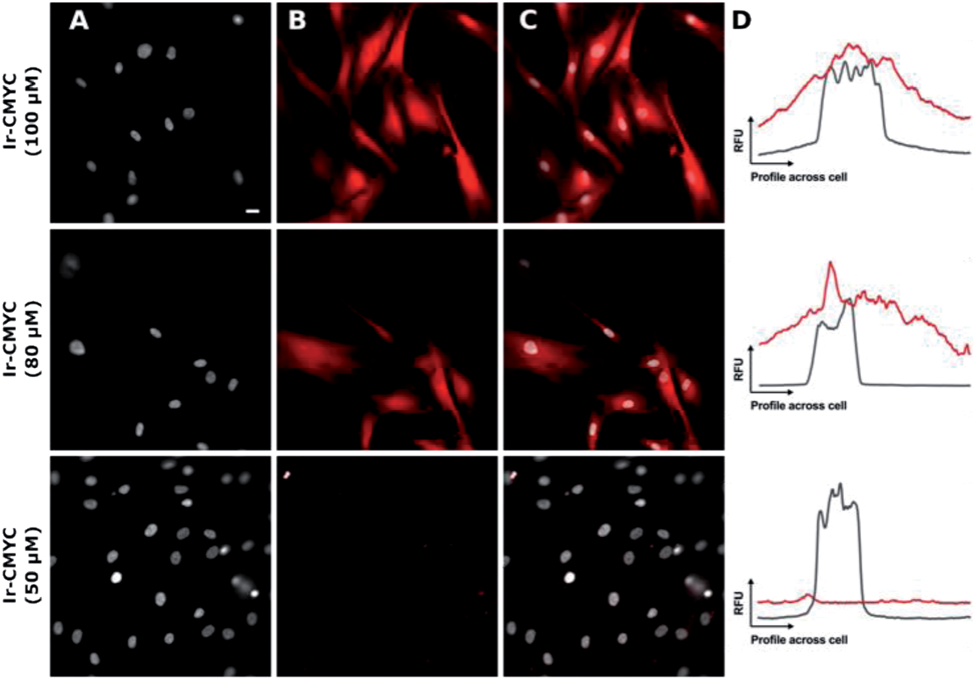
The addition of the c-Myc tag results in a significantly greater degree of nuclear co-localization. **Ir-CMYC** (red, (B)) was incubated with human fibroblast at a range of concentrations (50, 80 & 100 μM) for 18 hours prior to counterstaining with Hoechst 33342 (grey, (A)). Merged images (C) show nuclear co-localization at concentrations of 80 and 100 μM. Sample co-localization plots (D) show the intensity in relative fluorescent units (RFU) of **Ir-CMYC** (red) and Hoechst 33342 (grey) across a cell. Representative scale bar in (A) is equal to 7.5 μm, images acquired at (*λ*_ex_ = 480 nm; *λ*_em_ = 645 nm).

Having demonstrated that **Ir-PYR** does not localize in the cell nucleus, further imaging studies were then conducted on the peptide-functionalised analogue, **Ir-CMYC**. Again, a series of concentrations were used (1, 5, 20, 50, 80, 100 μM) for incubation with the cells. No uptake or staining was observed for 1–5 μM **Ir-CMYC**, but with increasing concentrations there was very clear uptake and staining evident (Fig. 5).

At 20–50 μM weak punctate staining was observed throughout the cytoplasm, and at 80–100 μM strong staining was seen throughout the cell, with clear nuclear localization. Co-localization experiments using Hoechst 33342 demonstrate that **Ir-CMYC** does enter and localize in the cell nucleus (Fig. 5). At 24 hours after **Ir-CMYC** incubation, there were no detectable changes in cellular morphology or mitochondrial activity (as monitored by a resazurin cell death assay) indicating no (or minimal) toxicity of **Ir-CMYC** to the cells.

**Fig. 6:**
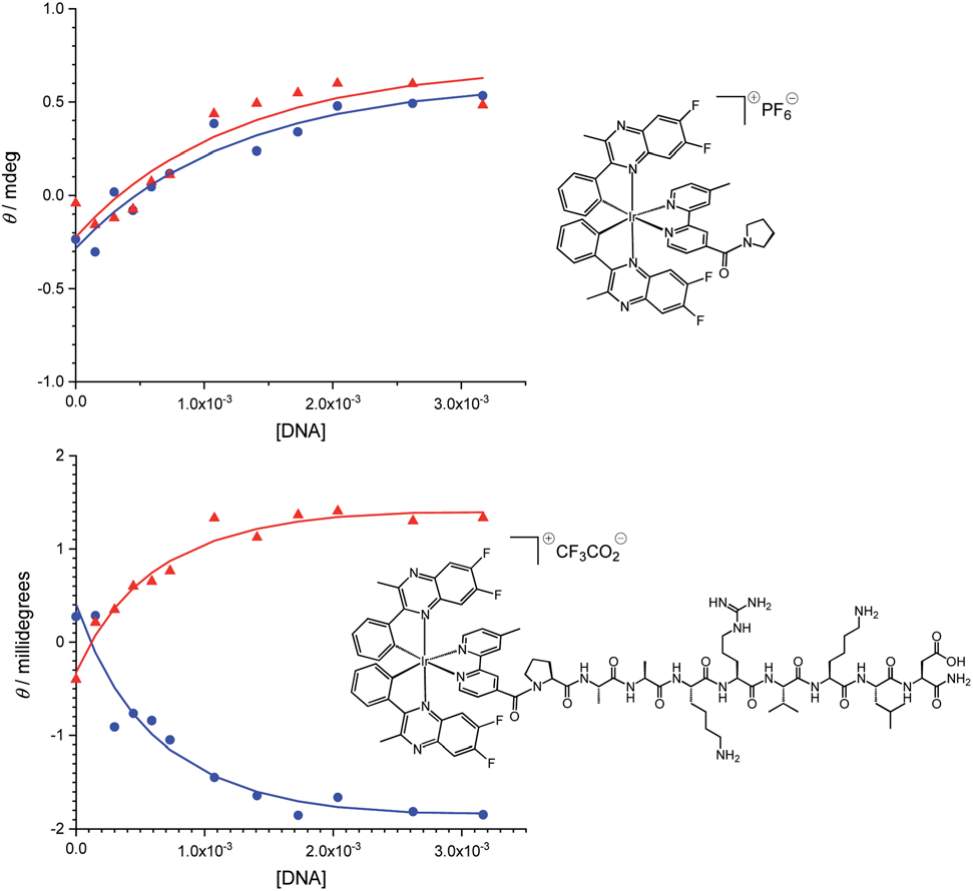
**Ir-PYR** and **Ir-CMYC** both interact weakly with DNA. Circular dichroism titration of **Ir-PYR** (top) with fish sperm DNA, 400 nm ([black circle]), 493 nm (▲); titration for **Ir-CMYC** (bottom), 373 nm ([black circle]), 493 nm (▲).

In view of the nuclear localisation of **Ir-CMYC**, we also considered the potential role that affinity to DNA may play. To explore DNA binding of these Ir(iii) complexes, UV-vis and circular dichroism titrations were carried out in buffer (25 mM MOPS, 50 mM NaCl, 1 mM EDTA) at 25 °C (Fig. S6, ESI†). To quantify the affinity of **Ir-PYR** for fish sperm DNA, titration curves at 400 and 493 nm were extracted from the circular dichroism data (Fig. 6). Similarly, for **Ir-CMYC**, titration curves at 373 and 493 nm were extracted from the circular dichroism data. The binding data were analysed globally (Table S1†) in terms of the multiple independent binding sites model, explicitly taking into account the dilution of the iridium complex during the titration.^28^ This analysis gave binding constants of (1.5 ± 0.7) × 10^3^ M^–1^ and (4.5 ± 1.0) × 10^3^ M^–1^ for **Ir-PYR** and **Ir-CMYC**, respectively (full fitting details in the ESI†). Although **Ir-CMYC** appears to bind a bit more strongly to DNA than **Ir-PYR**, the difference is less than an order of magnitude and both affinities are low. Indeed such low values are indicative of an interaction with DNA which is driven by electrostatics, suggesting that neither the c-Myc NLS or the cationic Ir(iii) complex moiety strongly interact with the DNA structure.

To investigate whether the NLS was cleaved off **Ir-CMYC** prior to translocation to the nucleus, an immunoassay was trialled using an antibody raised against c-Myc. Following the immunocytochemistry procedure which permeabilises the cell membrane, the red emission from **Ir-CMYC** was no longer evident in the cells using fluorescence microscopy imaging. This suggests that permeabilisation of the cell membrane causes **Ir-CMYC** to leak out of the cell consistent with not being bound to DNA.

Taken together, these results suggest that the fundamental differences in nuclear localisation observed between **Ir-PYR** and **Ir-CMYC** cannot be explained in terms of an affinity for DNA: the nuclear localisation of **Ir-CMYC** is facilitated by the PAAKRVKLD sequence.

## Conclusion

In summary, the synthesis, spectroscopic properties and cellular translocation characteristics of a deep red luminescent organometallic Ir(iii) complex conjugated with a c-Myc NLS have been described. The NLS conjugated complex is essentially non-toxic, which contrasts strongly with the non-peptide complex analogue, which induces mitochondrial dysfunction and is toxic ≥20 μM. The conjugated complex was demonstrated, *via* UV-vis and circular dichroism titrations, to have a very weak affinity for DNA (∼4.5 × 10^3^ M^–1^). Therefore, through a range of biological and spectroscopic studies it was demonstrated that the c-Myc nuclear localization sequence PAAKRVKLD promotes nuclear uptake of an organometallic iridium(iii) complex in human fibroblast cells.

## Experimental

### General considerations

All reagents and solvents were commercially available and were used without further purification if not stated otherwise. For the measurement of ^1^H, ^31^P, and ^13^C NMR spectra a Bruker Fourier^300^ (300 MHz), Bruker AVANCE HD III equipped with a BFFO SmartProbe™ (400 MHz) or Bruker AVANCE III HD with BBO Prodigy CryoProbe (500 MHz) was used. The obtained chemical shifts *δ* are reported in ppm and are referenced to the residual solvent signal. Spin–spin coupling constants *J* are given in Hz.

Low-resolution mass spectra were obtained by the staff at Cardiff University. High resolution mass spectral (HRMS) data were obtained on a Waters MALDI-TOF mx at Cardiff University or on a Thermo Scientific LTQ Orbitrap XL by the EPSRC UK National Mass Spectrometry Facility at Swansea University. IR spectra were obtained from a Shimadzu IR-Affinity-1S FTIR. Reference to spectroscopic data are given for known compounds. Analytical HPLC was performed using an Agilent 1260 infinity II equipped with a Zorbax SB-C18 2.1 × 50 mm column and an Agilent 6120 Quadrupole LC/MS detector over a 13 minute gradient elution. Preparatory HPLC was performed using a Dionex UltiMate 3000 equipped with a YMC-Triart Prep C18-S 250 × 19 mM column over a 55 min gradient elution. UV-vis studies were performed on a Shimadzu UV-1800 spectrophotometer as MeCN solutions (2.5 or 5 × 10^–5^ M). Photophysical data were obtained on a JobinYvon–Horiba Fluorolog spectrometer fitted with a JY TBX picosecond photodetection module as MeCN solutions. Emission spectra were uncorrected and excitation spectra were instrument corrected. The pulsed source was a Nano-LED configured for 459 nm output operating at 1 MHz. Luminescence lifetime profiles were obtained using the JobinYvon–Horiba FluoroHub single photon counting module and the data fits yielded the lifetime values using the provided DAS6 deconvolution software. Quantum yield measurements were obtained on aerated MeCN solutions of the complexes using [Ru(bpy)_3_](PF_6_)_2_ in aerated MeCN as a standard (*Φ* = 0.016).

### Human fibroblast primary cell culture

Human derived skin fibroblast cells lines were obtained from the Coriell Cell Repository (GM05399, Camden, NJ). Cells were cultured as monolayers in Dulbecco's modified Eagles medium (DMEM, Sigma, UK) supplemented with 10% fetal bovine serum (FBS, PAN-Biotech, Aidenbach, Germany) and 1% l-glutamine in a humidified incubator with 5% CO_2_ at 37 °C. For all imaging experiments low passage cells (P10-15) were seeded in 8-well chamber slides (Thistle, IB-80826) at a density of 15 000 cells in 250 μL of media.

### Cell staining with intracellular probes

To generate stock solutions, **Ir-CMYC** and **Ir-PYR** were resuspended in DMSO (final concentration 10 mM) and stored at –20 °C (it was noted that 3 freeze–thaws cycles did not appear to reduce the compound signal). Stock solutions were diluted to the stated concentrations in cell culture medium for incubation with fibroblasts. The equivalent amount of DMSO (<0.5% total volume) was added to controls for comparison. For nuclear visualisation cells were counterstained for 10 minutes with Hoechst 33342 diluted in cell culture media (1 μL mL^–1^, Thermo Fisher, UK). For non-live imaging, cells were fixed in cold 4% paraformaldehyde for 10 min at room temperature and rinsed with PBS. For live-cell colocalization studies, cells were incubated with **Ir-PYR** for 12 hours prior to counterstaining with Hoechst 33342, and either LysoTracker-green DND-26 (L7528, Thermo Fisher, UK), or CYTO-ID Autophagy detection kit 2.0 (175–0050, Enzo Life Sciences, UK), as per the manufacturer's instructions. Briefly, LysoTracker was diluted in cell culture media to a final concentration of 200 nM and left for 15 min at 37 °C. CYTO-ID was diluted by adding 2 μL of CYTO-ID green reagent in 1 mL of assay buffer and incubated for 30 min at 37 °C. After organelle staining, cells were washed twice in DPBS and immediately imaged in FluoroBrite DMEM (A1896701, Thermo Fisher, UK) supplemented with 10% FBS.

### Monitoring cellular metabolic activity

Human fibroblasts were plated at 10 000 cells per well of a 96-well plate (Cellbind) and treated with the indicated concentrations of **Ir-PYR**, **Ir-CMYC** or DMSO. As a measure of cell viability and metabolic activity, at 18 hours post compound addition culture medium was removed and replaced with culture medium containing 10 μg mL^–1^ resazurin diluted in DPBS (10% v/v). After a 4 hour incubation at 37 °C, fluorescence was determined using a Molecular Devices Spectramax Gemini EM plate reader (*λ*_ex_ = 445 nm; *λ*_em_ = 585 nm).

### Imaging and localisation analysis

All widefield fluorescent microscopy was performed using a Zeiss 35 fitted with Cairn Optospin filter wheels at excitation and emission ports, an Orca flash 4.0/4.2, sCMOS camera, Exfo xCite multiband lightsource, and Micro-Manager software.^29^

For confocal microscopy, live cells were imaged on an inverted Leica DMIRE2 with TCS SP2 AOBS confocal system using Leica AF software in combination with a 405 nm diode laser (Hoechst), a 458/476/488/514 nm argon multiline (LysoTracker and CYTO-ID), and a 594 nm HeNe (**Ir-PYR**). Images were captured using a 40× oil-immersion objective coupled to an additional 2× magnifying lens.^30^ During imaging, cells were maintained at 37 °C using an environmental chamber.

To generate intensity colocalization plots, images were imported into Fiji as separate channels,^31^ and a line drawn across an individual cell. Plot profile was used to generate intensity of gray values across the ROI. Plots using data from both channels was generated using Prism version 8.1.0 (GraphPad Software, La Jolla California, USA). All experimental cell data was repeated twice (*n* = 2), with three technical replicates per condition used in the individual experiment.

### Monitoring cellular metabolic activity

Human fibroblasts were plated at 10 000 cells per well of a 96-well plate and treated with the various concentrations of **Ir-PYR**, **Ir-CMYC** (5, 20, 80, 50, 100 μM) or the highest volume of equivalent of DMSO only. As a measure of cell viability and metabolic activity, at 18 hours post compound addition culture medium was removed and replaced with culture medium containing 10 μg mL^–1^ resazurin diluted in DPBS (10% v/v). Metabolically active cells reduce resazurin (blue and non-fluorescent) to resorufin (pink and fluorescent). After a 4 hour incubation at 37 °C, fluorescence was determined using a Molecular Devices Spectramax Gemini EM plate reader (*λ*_ex_ = 530 nm; *λ*_em_ = 580 nm).

### DNA-binding experiments

MOPS buffer was prepared by dissolving MOPS (3-(*N*-morpholino)propanesulfonic acid, CAS[1132-61-2]), NaCl, and EDTA (ethylenediaminetetraacetic acid disodium salt dihydrate, CAS[6381-92-6]) (all obtained from Fisher and used as supplied) in deionised water (Elga Purelab Flex), adjusting the pH to 7.0 using a NaOH solution (pH of the buffer was determined using a Hanna Instruments pH210 microprocessor pH meter with a VWR simple junction universal combined pH/reference electrode) and making up the solution to 0.5 litre.

A stock solution of fish sperm DNA was prepared by dissolving approximately 0.1 g of fish sperm DNA in 10 mL of the buffer. The resulting solution was dialysed (3.5 kDa MWCO, Visking, Medicell International Ltd) against 0.5 litres of buffer. Following dialysis, the DNA concentration was determined using UV-visible spectroscopy (Shimadzu UV-1800 spectrophotometer) using an extinction coefficient *ε*_260nm_ = 12 800 M(bp)^–1^ cm^–1^.^32^

Stock solutions containing 10 mM **Ir-PYR** or **Ir-CMYC** were made as follows. For **Ir-PYR**, 1.01 mg was weighed out and dissolved in 104.1 μL of DMSO. For **Ir-CMYC**, 0.6 mg was weighed out and dissolved in 31.6 μL of DMSO.

Circular dichroism titrations were carried out using an Applied Photophysics Chirascan spectrophotometer thermostated at 25 °C. First, 2500 μL of buffer was placed in a 1 cm pathlength quartz cuvette (Hellma) and a spectrum was recorded between 700 and 230 nm. Next, between 10 and 15 μL (typically 12.5 μL) of the stock solution of the iridium complex in DMSO was added to the buffer and a spectrum was recorded. Subsequently, 5 aliquots of 20 μL, 4 aliquots of 50 μL and 2 aliquots of 100 μL (*i.e.* a cumulative added volume of 500 μL) of the DNA stock solution were added and a spectrum was recorded after every addition.

### DNA binding – data analysis results

Spectra and titrations were plotted in OriginLab Origin 2019b. The titration data were analysed globally for each complex using an in-house written version of the multiple independent binding sites model which also explicitly takes ligand concentrations into account.^28^ Exploratory data analysis showed that binding affinity and binding site size could not be determined separately from this data, as is usual for relatively weak binding. The binding site size (*n*) was therefore set to three base pairs for both complexes. The molar ellipticity of the free ligand (*θ*_m,free/mdeg_) was restricted to 0, *i.e.* no CD signal in the absence of DNA while the background ellipticity at the wavelength of interest (*θ*_background,*x* nm_) was an optimisable parameter to address the effects of noise on the data. The data analysis thus provided optimised values for the equilibrium constant (*K*), the changes in molar ellipticity upon binding at the wavelengths of interest (Δ*θ*_m,*x* nm_) and the background ellipticities at the wavelengths of interest (*θ*_background,*x* nm_). These values are summarised in Table S1.†

## Conflicts of interest

There are no conflicts to declare.

## Supplementary Material

Supplementary information

## References

[cit1] von Heijne G., Owji H., Nezafat N., Negahdaripour M., Hajiebrehami A. (1990). J. Membr. Biol..

[cit2] Marfori M., Mynott A., Ellis J. J., Mehdi A. M., Saunders N. F. W., Curmi P. M., Forwood J. K., Boden M., Kobe B. (2011). Biochim. Biophys. Acta.

[cit3] Dingwall C., Laskey R. A., Lange A., Mills R. E., Lange C. J., Stewart M., Devine S. E., Corbett A. H. (1991). Trends Biochem. Sci..

[cit4] Makkerh J. P. S., Dingwall C., Laskey R. A. (1996). Curr. Biol..

[cit5] Dang C. V., Lee W. M. (1988). Mol. Cell. Biol..

[cit6] Finver S. N., Nishikura K., Finger L. R., Haluska F. G., Finan J., Nowell P. C., Croce C. M. (1988). Proc. Natl. Acad. Sci. U. S. A..

[cit7] Stine Z. E., Walton Z. E., Altman B. J., Hsieh A. L., Dang C. V., Dang C. V. (2015). Cancer Discovery.

[cit8] Chen H., Lui H., Qing G. (2018). Signal Transduction Targeted Ther..

[cit9] Singh S., Gramolini A. O. (2009). BMC Cell Biol..

[cit10] Niopek D., Benzinger D., Roensch J., Draebing T., Wehler P., Eils R., di Ventura B. (2014). Nat. Commun..

[cit11] Ray M., Tang R., Jiang Z., Rotello V. M. (2015). Bioconjugate Chem..

[cit12] Thomas J. A., Baggaley E., Weinstein J. A., Williams J. A. G., Zamora A., Vigueras G., Rodriguez V., Dolores Santana M., Ruiz J., Sreedharan S., Gill M. R., Garcia E., Saeed H. K., Robinson D., Byrne A., Cadby A., Keyes T. E., Smythe C., Pellett P., de la Serna J. B., Thomas J. A., Thorp-Greenwood F. L., Balasingham R. G., Coogan M. P., Lo K. K., Louie M., Sze K., Lau J., Baggaley E., Gill M. R., Green N. H., Turton D., Sazanovich I. V., Botchway S. W., Smythe C., Haycock J. W., Weinstein J. A., Baggaley E., Botchway S. W., Haycock J. W., Morris H., Sazanovich I. V., Williams J. A. G., Weinstein J. A., Lo K. K.-W., Chung C.-K., Lee T. K.-M., Lui L.-H., Tsang K. H.-K., Zhu N., Amoroso A. J., Coogan M. P., Dunne J. E., Fernández-Moreira V., Hess J., Hayes A. J., Lloyd D., Millet C., Pope S. J. A., Williams C., Langdon-Jones E. E., Symonds N. O., Yates S. E., Hayes A. J., Lloyd D., Williams R., Coles S. J., Horton P. N., Pope S. J. A., Langdon-Jones E. E., Williams C. F., Hayes A. J., Lloyd D., Coles S. J., Horton P. N., Groves L. M., Pope S. J. A., Langdon-Jones E. E., Jones A. B., Williams C. F., Hayes A. J., Lloyd D., Mottram H. J., Pope S. J. A., Balasingham R. G., Thorp-Greenwood F. L., Williams C. F., Coogan M. P., Pope S. J. A., Li C., Yu M., Sun Y., Wu Y., Huang C., Li F., Lee P.-K., Law W. H.-T., Liu H.-W., Lo K. K.-W., Jana A., Baggaley E., Amoroso A. J., Ward M. D. (2015). Chem. Soc. Rev..

[cit13] Yang G.-J., Wang W., Mok S. W. F., Wu C., Law B. Y. K., Miao X.-M., Wu K.-J., Zhong H.-J., Wong C.-Y., Wong V. K. W., Ma D.-L., Leung C.-H., Vellaisamy K., Li G., Weng W., Leung C.-H., Ma D.-L., Zhang S., Hosaka M., Yoshihara T., Negishi K., Iida Y., Tobita S., Takeuchi T. (2018). Angew. Chem., Int. Ed..

[cit14] Coogan M. P., Fernandez-Moreira V., Lo K. K., Le T. K. M., Lo J. S. Y., Poon W. L., Cheng S. H. (2014). Chem. Commun..

[cit15] Viola-Villegas N., Rabideau A. E., Cesnavicious J., Zubieta J., Doyle R. P. (2014). Dalton Trans..

[cit16] He H., Morely J. E., Silva-Lopez E., Bottenus B., Montajano M., Fugate G. A., Twamley B., Benny P. D., La Bella R., Garcia-Garayoa E., Bahler M., Blauenstein P., Schibli R., Conrath P., Tourwe D., Schubiger P. A., Polyakov V., Sharma V., Dahlheimer J. L., Pica C. M., Luker G. D., Picwnica-Worms D. (2009). Bioconjugate Chem..

[cit17] Neugebauer U., Pellegrin Y., Devocelle M., Forster R. J., Signac W., Moran N., Keyes T. E. (2008). Chem. Commun..

[cit18] Cosgrave L., Devocelle M., Forster R. J., Keyes T. E. (2010). Chem. Commun..

[cit19] Martin A., Byrne A., Burke C. S., Forster R. J., Keyes T. E. (2014). J. Am. Chem. Soc..

[cit20] Blackmore L., Moriarty R., Dolan C., Adamson K., Forster R. J., Devocelle M., Keyes T. E. (2013). Chem. Commun..

[cit21] Burke C. S., Byrne A., Keyes T. E. (2018). J. Am. Chem. Soc..

[cit22] Marcelis L., Kajou S., Ghesquiere J., Fettweis G., Coupienne I., Lartia R., Surin M., Defrancq E., Piette J., Moucheron C., Kirsch-De Mesmaeker A. (2016). Eur. J. Inorg. Chem..

[cit23] Archer S. A., Raza A., Droge F., Robertson C., Auty A. J., Chekulaev D., Weinstein J. A., Keane T., Meijer A. J. H. M., Haycock J. W., MacNeil S., Thomas J. A., Stacey O. J., Pope S. J. A., Colombo A., Fontani M., Dragometti C., Roberto D., Williams J. A. G., Scotto di Perrotolo R., Casagrande F., Barozzi S., Polo S. (2019). Chem. Sci..

[cit24] Langdon-Jones E. E., Hallett A. J., Routledge J. D., Crole D. A., Ward B. D., Platts J. A., Pope S. J. A. (2013). Inorg. Chem..

[cit25] Smith R. A., Stokes E. C., Langdon-Jones E. E., Platts J. A., Kariuki B. M., Hallett A. J., Pope S. J. A. (2013). Dalton Trans..

[cit26] Phillips K. A., Stonelake T. M., Chen K., Hou Y., Zhao J., Coles S. J., Horton P. N., Keane S. J., Stokes E. C., Fallis I. A., Hallett A. J., O'Kell S., Beames J. M., Pope S. J. A. (2018). Chem.–Eur. J..

[cit27] Yang J., Zhao J.-X., Cao Q., Hao L., Zhou D., Gan Z., Ji L.-N., Mao Z.-W., Tian M., Li J., Zhang S., Guo L., He X., Kong D., Zhang H., Liu Z., Li J. J., Tian M., Tian Z., Zhang S., Yan C., Shao C., Liu Z., Chen M.-H., Wang F.-X., Cao J.-J., Tan C.-P., Ji L.-N., Mao Z.-W., He L., Li Y., Tan C.-P., Ye R.-R., Chen M.-H., Cao J.-J., Ji L.-N., Mao Z.-W. (2017). ACS Appl. Mater. Interfaces.

[cit28] Hahn L., Buurma N. J., Gade L. H. (2016). Chem.–Eur. J..

[cit29] Edelstein A. D., Tsuchida M. A., Amodaj N., Pinkard H., Vale R. D., Stuurman N. (2014). Journal of Biological Methods.

[cit30] Lloyd-Evans E., Morgan A. J., He X., Smith D. A., Elliot-Smith E., Sillence D. J., Churchill G. C., Schuchman E. H., Galione A., Platt F. M. (2008). Nat. Med..

[cit31] Schindelin J., Arganda-Carreras I., Frise E., Kaynig V., Longair M., Pietzsch T., Preibisch S., Rueden C., Saalfeld S., Schmid B., Tinevez J.-Y., White D. J., Hartenstein V., Eliceiri K., Tomancak P., Cardona A. (2019). Nat. Methods.

[cit32] Mullice L. A., Laye R. H., Harding L. P., Buurma N. J., Pope S. J. A. (2008). New J. Chem..

