# Supplementary material for: Targeted cell imaging properties of a deep red luminescent iridium(iii) complex conjugated with a c-Myc signal peptide
Source: Chem Sci. 2020 Jan 8;11(6):1599–606. doi: 10.1039/c9sc05568a (PMC7069228; doi:10.1039/c9sc05568a)
Supplement: Supplementary file 1 [file SC-011-C9SC05568A-s001.pdf]

Electronic Supplementary Material (ESI) for Chemical Science.
This journal is © The Royal Society of Chemistry 2019

Targeted Cell Imaging Properties of a Deep Red Luminescent Iridium(III) Complex Conjugated With a c-Myc Signal Peptide

Adam H. Day,[a] Martin H. Ubler,[a] Hannah L. Best,[b] Emyr Lloyd-Evans,[b] Robert J. Mart,[a] Rudolf K. Allemann,[a] Eman A.H. Al-Watter,[a] Niklaas J. Buurma, [a] Ian A. Fallis,[a] and Simon J.A. Pope*[a]

[a]School of Chemistry, Main Building, Cardiff University, Cardiff, UK CF10 3AT;
[b]School of Biosciences, Cardiff University, Cardiff, UK.

Supplementary Information

Contents

Experimental details	page 2
Synthesis of the PAAKRVKLD peptide	page 4
Synthesis of ligands and complexes	page 7
Figure S1	page 9
Figure S2	page 10
Figure S3	page 11
Figure S4	page 12
Figure S5	page 12
Figure S6	page 13
Table S1	page 13
References	page 14

Experimental

General

All reagents and solvents were commercially available and were used without further purification if not stated otherwise. For the measurement of ^{1}H, ^{31}P, and ^{13}C NMR spectra a Bruker Fourier^{300} (300 MHz), Bruker AVANCE HD III equipped with a BFFO SmartProbe™ (400 MHz) or Bruker AVANCE III HD with BBO Prodigy CryoProbe (500 MHz) was used. The obtained chemical shifts δ are reported in ppm and are referenced to the residual solvent signal. Spin-spin coupling constants *J* are given in Hz.

Low-resolution mass spectra were obtained by the staff at Cardiff University. High-resolution mass spectra were carried out at the EPSRC National Mass Spectrometry Facility at Swansea University. High resolution mass spectral (HRMS) data were obtained on a Waters MALDI-TOF mx at Cardiff University or on a Thermo Scientific LTQ Orbitrap XL by the EPSRC UK National Mass Spectrometry Facility at Swansea University. IR spectra were obtained from a Shimadzu IR-Affinity-1S FTIR. Reference to spectroscopic data are given for known compounds. UV-Vis studies were performed on a Shimadzu UV-1800 spectrophotometer as MeCN solutions (2.5 or 5 × 10^{-5} M). Photophysical data were obtained on a JobinYvon–Horiba Fluorolog spectrometer fitted with a JY TBX picosecond photodetection module as MeCN solutions. Emission spectra were uncorrected and excitation spectra were instrument corrected. The pulsed source was a Nano-LED configured for 459 nm output operating at 1 MHz. Luminescence lifetime profiles were obtained using the JobinYvon–Horiba FluoroHub single photon counting module and the data fits yielded the lifetime values using the provided DAS6 deconvolution software. Quantum yield measurements were obtained on aerated MeCN solutions of the complexes using $[Ru(bpy)_3](PF_6)_2$ in aerated MeCN as a standard (Φ = 0.016).

Human fibroblast primary cell culture

Human derived skin fibroblast cells lines were obtained from the Coriell Cell Repository (GM05399, Camden, NJ). Cells were cultured as monolayers in Dulbecco's modified Eagles medium (DMEM, Sigma, UK) supplemented with 10% fetal bovine serum (FBS, PAN-Biotech, Aidenbach, Germany) and 1% L-glutamine in a humidified incubator with 5% CO_2 at 37°C. For all imaging experiments low passage cells (P10-15) were seeded in 8-well chamber slides (Thistle, IB-80826) at a density of 15,000 cells in 250 uL of media.

Cell staining with intracellular probes

To generate stock solutions, **Ir-CMYC** and **Ir-PYR** were resuspended in DMSO (final concentration 10 mM) and stored at -20°C (it was noted that 3 freeze-thaws cycles did not appear to reduce the compound signal). Stock solutions were diluted to the stated concentrations in cell culture medium for incubation with fibroblasts. The equivalent amount of DMSO (<0.5% total volume) was added to controls for comparison. For nuclear visualisation cells were counterstained for 10 minutes with Hoescht 33342 diluted in cell culture media (1 uL/mL, Thermo Fisher, UK). For non-live imaging, cells were fixed in cold 4% paraformaldehyde for 10 mins at room temperature and rinsed with PBS. For live-cell colocalization studies, cells were incubated with **Ir-PYR** for 12 hours prior counterstaining with Hoescht 33342, and either LysoTracker-geen DND-26 (L7528, Thermo Fisher, UK), or CYTO-ID Autophagy detection kit 2.0 (175-0050, Enzo Life Sciences, UK), as per the manufacturer's instructions. Briefly, LysoTracker was diluted in cell culture media to a final concentration of 200 nM and left for 15 mins at 37 °C. CYTO-ID was diluted by adding 2 uL of CYTO-ID green reagent in 1 mL of assay buffer and incubated for 30 mins at 37°C. After organelle staining, cells were washed twice in DPBS and immediately imaged in FluoroBrite DMEM (A1896701, Thermo Fisher, UK) supplemented with 10% FBS.

Monitoring cellular metabolic activity

Human fibroblasts were plated at 10,000 cells/well of a 96-well plate (Cellbind) and treated with the indicated concentrations of **Ir-PYR**, **Ir-CMYC** or DMSO. As a measure of cell viability and metabolic activity, at 18-hours post compound addition culture medium was removed and replaced with culture medium containing 10 μg/mL resazurin diluted in DPBS (10% V/V). After a 4 hour incubation at 37°C, fluorescence was determined using a Molecular Devices Spectramax Gemini EM plate reader (λ_{ex} = 445 nm; λ_{em} = 585 nm).

Imaging and localisation analysis

All widefield fluorescent microscopy was performed using a Zeiss 35 fitted with Cairn Optospin filter wheels at excitation and emission ports, an Orca flash 4.0/4.2, sCMOS camera, Exfo xCite multiband lightsource, and Micro-Manager software.[1]

For confocal microscopy, live cells were imaged on an inverted Leica DMIRE2 with TCS SP2 AOBS confocal system using Leica AF software in combination with a 405 nm diode laser (Hoescht), a 458/476/488/514 nm Argon multiline (LysoTracker and CYTO-ID), and a

594 nm HeNe (**Ir-PYR**). Images were captured using a 40 x oil-immersion objective coupled to an additional 2x magnifying lens. During imaging, cells were maintained at 37°C using an environmental chamber.

To generate Intensity colocalization plots, images were imported into Fiji as separate channels,[2] and a line drawn across an individual cell. Plot profile was used to generate intensity of gray values across the ROI. Plots using data from both channels was generated using Prism version 8.1.0 (GraphPad Software, La Jolla California, USA). All experimental cell data was repeated twice (n = 2), with three technical replicates per condition used in the individual experiment.

Monitoring cellular metabolic activity (supplementary method)

Human fibroblasts were plated at 10,000 cells/well of a 96-well plate and treated with the various concentrations of **Ir-PYR**, **Ir-CMYC** (5, 20, 80, 50, 100 μM) or the highest volume of equivalent of DMSO only. As a measure of cell viability and metabolic activity, at 18-hours post compound addition culture medium was removed and replaced with culture medium containing 10 μg/mL resazurin diluted in DPBS (10% V/V). Metabolically active cells reduce resazurin (blue and non-fluorescent) to resorufin (pink and fluorescent). After a 4-hour incubation at 37°C, fluorescence was determined using a Molecular Devices Spectramax Gemini EM plate reader (λ_{ex} = 530 nm; λ_{em} = 580 nm).

DNA-binding experiments

MOPS buffer was prepared by dissolving MOPS (3-(N-morpholino)propanesulfonic acid, CAS[1132-61-2]), NaCl, and EDTA (ethylenediaminetetraacetic acid disodium salt dihydrate, CAS[6381-92-6]) (all obtained from Fisher and used as supplied) in deionised water (Elga Purelab Flex), adjusting the pH to 7.0 using a NaOH solution (pH of the buffer was determined using a Hanna Instruments pH210 microprocessor pH meter with a VWR simple junction universal combined pH/reference electrode) and making up the solution to 0.5 litre.

A stock solution of fish sperm DNA was prepared by dissolving approximately 0.1 g of fish sperm DNA in 10 mL of the buffer. The resulting solution was dialysed (3.5k Da MWCO, Visking, Medicell International Ltd) against 0.5 litres of buffer. Following dialysis, the DNA concentration was determined using UV-visible spectroscopy (Shimadzu UV-1800 spectrophotometer) using an extinction coefficient ε_{260nm} = 12800 $M(bp)^{-1}$ cm^{-1}.[3]

Stock solutions containing 10 mM **Ir-PYR** or **Ir-CMYC** were made as follows. For **Ir-PYR**, 1.01 mg was weighed out and dissolved in 104.1 µL of DMSO. For **Ir-CMYC**, 0.6 mg was weighed out and dissolved in 31.6 µL of DMSO.

Circular dichroism titrations were carried out using an Applied Photophysics Chirascan spectrophotometer thermostated at 25 °C. First, 2500 uL of buffer was placed in a 1 cm pathlength quartz cuvette (Hellma) and a spectrum was recorded between 700 and 230 nm. Next, between 10 and 15 uL (typically 12.5 uL) of the stock solution of the iridium complex in DMSO was added to the buffer and a spectrum was recorded. Subsequently, 5 aliquots of 20 µL, 4 aliquots of 50 µL and 2 aliquots of 100 µL (i.e. a cumulative added volume of 500 µL) of the DNA stock solution were added and a spectrum was recorded after every addition.

DNA binding – data analysis results

Spectra and titrations were plotted in OriginLab Origin 2019b. The titration data were analysed globally for each complex using an in-house written version of the multiple independent binding sites model which also explicitly takes ligand concentrations into account.[4] Exploratory data analysis showed that binding affinity and binding site size could not be determined separately from this data, as is usual for relatively weak binding. The binding site size (n) was therefore set to three base pairs for both complexes. The molar ellipticity of the free ligand ($\theta_{m,free}$ / mdeg) was restricted to 0, i.e. no CD signal in the absence of DNA while the background ellipticity at the wavelength of interest ($\theta_{background,\ x\ nm}$) was an optimisable parameter to address the effects of noise on the data. The data analysis thus provided optimised values for the equilibrium constant (K), the changes in molar ellipticity upon binding at the wavelengths of interest ($\Delta\theta_{m,\ x\ nm}$) and the background ellipticities at the wavelengths of interest ($\theta_{background,\ x\ nm}$). These values are summarised in Table S1.

Synthesis of the PAAKRVKLD peptide sequence

Fmoc-solid phase peptide synthesis was performed using Rink Amide resin (0.19 mmol g^{-1} loading, CEM Corporation) at 0.1 mmol scale using standard techniques. Coupling was performed using 5.0 eq. HBTU (*O*-(benzotriazol-1-yl)-*N,N,N',N'*-tetramethyluronium hexafluorophosphate), 5.0 eq. HOBT (1-hydroxybenzotriazole), DIPEA (*N,N*-diisopropylethylamine) and 4.0 eq. of amino acid. Single coupling was performed for 60 min, double coupling was carried out for 45 min per cycle for arginine and valine and their direct successive amino acids. Fmoc-deprotection was achieved by exposing the resin to 3 mL of a

10% (*w*/*v*) piperazine in NMP (1-methyl-2-pyrrolidinone)/ethanol (9:1 (*v*/*v*)) + 0.1 M HOBt (*O*-(benzotriazol-1-yl)-*N,N,N′,N′*-tetramethyluronium hexafluorophosphate) for 15 min, twice.

Synthesis of Ir-CMYC

The crude peptide was subsequently coupled to 4.9 eq. of **Ir-COOH** (see below) overnight using the coupling conditions described. The peptide-metal complex was cleaved off the resin by stirring it in 92.5 % (*v*/*v*) TFA (trifluoroacetic acid), 2.5 % (*v*/*v*) water, 2.5 % (*v*/*v*) DODT (2,2′-(ethylenedioxy)diethanethiol), 2.5 % (*v*/*v*) TIS (triisopropylsilane) for 2 h at room temperature. The peptide-metal complex was then purified by HPLC (YMC-Triart Prep C18-S, 250 x 10 mm) using a linear gradient of 10 % to 95 % acetonitrile-water (+0.1% trifluoroacetic acid) over 60 min. The product eluted at 28.4 min and the molecular composition of the peptides was confirmed by LC-MS. Lyophilized peptide was used for the subsequent characterization and the biological testing.

Synthesis of ligands and complexes

The 6,7-difluoro-2-methyl-3-phenylquinoxaline (L) and the corresponding iridium(III) dimer $[\{Ir(L)_2(\mu\text{-}Cl)\}_2]$ were synthesised as previously described.[5]

Synthesis of 4'-methyl-(2.2'-bipyridine)-4-(pyrrolidin-1-yl)methanone

4-methyl-2,2,-bipyridil-4'-carboxylic acid (200 mg, 0.93 mmol) was suspended in thionyl chloride (20 mL) and heated at reflux over 16 hours. Once allowed to cool, solvent was remove *in vacuo* yielding the intermediate acyl chloride as an off-white solid. Pyrrolidine (200 mg, 2.80 mmol) was dissolved in acetonitrile (5 mL), which was then added with stirring to the dry acyl chloride intermediate, taking care to re-suspend and dissolve all intermediate prior to stirring at room temperature for 16 hours. Water (150 mL) was then carefully added to the reaction mixture, and the product extracted into dichloromethane (3 x 50 mL). The product was back-extracted into saturated citric acid (3 x 100 mL) which was then basified through the addition of sodium hydroxide (10 M). The product was then re-extracted into dichloromethane (3 x 50 mL) before drying with magnesium sulfate and removal of solvent *in vacuo* to yield the product as a tan coloured oil (yield = 170 mg, 68%). ^{1}H NMR (400 MHz, $CDCl_3$) δ 8.72 (dd, *J* = 4.9, 0.7 Hz, 1H), 8.50 (d, *J* = 4.9 Hz, 1H), 8.46 (dd, *J* = 1.5, 0.8 Hz, 1H), 7.38 (dd, *J* = 4.9, 1.6 Hz, 1H), 7.13 (ddd, *J* = 5.0, 1.6, 0.7 Hz, 1H), 3.64 (t, *J* = 6.9 Hz, 2H), 3.43 (t, *J* = 6.6 Hz, 2H), 2.00 – 1.84 (m, 4H). ^{13}C{^{1}H} NMR (126 MHz, $CDCl_3$) δ 167.5, 167.3, 156.8, 156.0, 155.3, 149.9,

149.7, 149.2, 148.4, 145.8, 145.7, 125.15, 122.1, 121.7, 121.3, 118.7, 77.5, 77.5, 77.4, 76.9, 49.3, 46.3, 46.2, 26.4, 24.5, 21.3. LRMS (ES+) found *m/z* = 267.14 for $[M+H]^+$. HRMS (NSI-FTMS +): expected *m/z* = 268.1444 for $[M+H]^+$; found *m/z* = 268.1448.

Synthesis of Ir-COOH

$[\{Ir(L)_2(\mu\text{-}Cl)\}_2]$ dimer (282 mg, 0.19 mmol) and 4-methyl-2,2,-bipyridil-4'-carboxylic acid (75 mg, 0.35 mmol) were suspended in 2-methoxyethanol and heated at reflux for 16 hours. Once allowed to cool, water (40 mL) was added, followed by the addition of saturated aqueous ammonium hexafluorophosphate (10 mL) resulting in the precipitation of the product. The product was collected by filtration, dissolved in dichloromethane (5 mL) and precipitated with diethyl ether to yield the product as a ruby coloured solid (yield = 215 mg, 61%). ^{1}H NMR (400 MHz, $CDCl_3$) δ 8.31 (d, *J* = 0.9 Hz, 1H), 8.26 – 8.21 (m, 3H), 8.18 (d, *J* = 0.5 Hz, 1H), 8.02 – 7.99 (m, 1H), 7.84 – 7.80 (m, 1H), 7.69 (s, *J* = 4.5 Hz, 1H), 7.53 (ddd, *J* = 18.6, 10.3, 8.4 Hz, 2H), 7.00 – 6.88 (m, 5H), 6.61 – 6.54 (m, 2H), 6.50 – 6.45 (m, 2H), 3.07 (d, *J* = 4.4 Hz, 6H), 2.21 (s, *J* = 4.6 Hz, 3H) ppm. $^{13}C\{^1H\}$ NMR (101 MHz, $CDCl_3$) δ 170.2, 170.1, 161.4, 160.3, 159.0, 158.9, 158.5, 158.2, 153.1, 152.0, 149.7, 149.6, 142.8, 140.5, 136.70, 136.6,,136.3, 134.7, 133.2, 131.1, 128.6, 128.5, 120.7, 120.5, 116.9, 116.6, 83.5, 31.9, 25.4, 19.9 ppm. LRMS (ES+) found *m/z* = 917.18 for $[M+H]^+$. HRMS (NSI-FTMS +): expected *m/z* = 917.1836 for $[M+H]^+$; found *m/z* = 917.1822. FT IR ν_{max} (solid / cm^{-1}) 419, 432, 442, 457, 473, 515, 557, 586, 638, 660, 669, 687, 741, 758, 793, 839, 876, 997, 1034, 1167, 1198, 1233, 1254, 1296, 1333, 1375, 1420, 1449, 1503, 1531, 1558, 1578, 1618, 1670, 2359, 2976, 3275 (OH). UV-vis (water) λ_{max} / nm (ε / $dm^3\ mol^{-1}\ cm^{-1}$) 468 (3448), 370 (19144), 263 (100000) nm.

Synthesis of Ir-PYR

4'-methyl-(2.2'-bipyridine)-4-(pyrrolidin-1-yl)methanone (22 mg, 0.082 mmol) and the $[\{Ir(L)_2(\mu\text{-}Cl)\}_2]$ dimer (61 mg, 0.041 mmol) were suspended in 2-methoxyethanol with a small amount of ammonium hexafluorophosphate (~100 mg) and heated at reflux over 16 hours. Once allowed to cool, water (40 mL) was added resulting in the precipitation of the product. The product was collected by filtration, dissolved in dichloromethane (2 mL) and purified by column chromatography using a gradient of 99:1 to 9:1 dichloromethane:methanol as the eluent to yield the product **Ir-PYR** as a crimson coloured solid (yield = 27 mg, 29%). ^{1}H NMR (500 MHz, $CDCl_3$) δ_H 8.43 (d, *J* = 8.2 Hz, 1H), 8.38 (d, 8.2 Hz, 1H), 8.40 – 8.36 (m, 1H), 8.40 – 8.36 (m, 1H), 8.35 – 8.31 (m, 1H), 8.35 – 8.30 (m, 1H), 8.28 – 8.21 (m, 1H), 8.21 – 8.15 (m, 1H), 7.96 – 7.91 (m, 1H), 7.78 – 7.69 (m, 2H), 7.58 (d, *J* = 5.9 Hz, 1H), 7.34 – 7.25 (m, 3H,

occluded by solvent peak), 7.05 (app. t, J = 9.4 Hz, 1H), 6.94 – 6.85 (m, 3H), 6.56 (d, J = 7.1Hz, 1H), 6.47 (d, J = 7.1Hz, 1H), 3.67 – 3.46 (m, 3H), 3.36 (s, 3H), 3.33 (s, 3H), 3.21 – 3.12 (m, 1H), 2.52 (s, 3H), 2.08 – 1.98 (m, 2H), 1.97 – 1.81 (m, 2H). $^{13}C\{^1H\}$ NMR (101 MHz, $CDCl_3$) δ_C 163.1, 162.7, 162.7, 155.2, 153.3, 153.2, 151.9, 151.4, 151.3, 147.9, 146.1, 144.7, 143.0, 142.8, 134.0, 133.7, 130.5 (d, $^2J_{CF}$ = 31.2 Hz) 129.9 (d, $^2J_{CF}$ = 44.7 Hz), 128.3, 126.1, 124.7, 122.7, 122.6, 122.0, 121.9, 115.0, 114.8, 110.1, 109.6, 109.6, 45.7, 30.9, 28.68, 28.7, 28.6, 28.6, 28.6, 28.5, 28.4, 28.3, 28.0, 26.7, 26.6, 25.1, 23.1, 21.7, 20.3, 13.1, 12.8. LRMS found m/z = 968.24 for $[M]^+$. HRMS expected m/z = 968.2445 for $[M]^+$; found m/z = 968.2446. FT IR ν_{max} (solid / cm^{-1}) = 403, 413, 419, 459, 505, 557, 586, 637, 667, 687, 700, 729, 756, 767, 793, 837, 878, 995, 1034, 1070, 1103, 1126, 1146, 1165, 1194, 1233, 1254, 1296, 1331, 1364, 1373, 1418, 1441, 1501, 1531, 1547, 1578, 1595, 1628, 2342, 2367, 2878, 2972, 3051. UV-vis (water) λ_{max} / nm (ε / $dm^3\ mol^{-1}\ cm^{-1}$) = 475 (3300), 370 (15500), 263 (99750) nm.

Figures

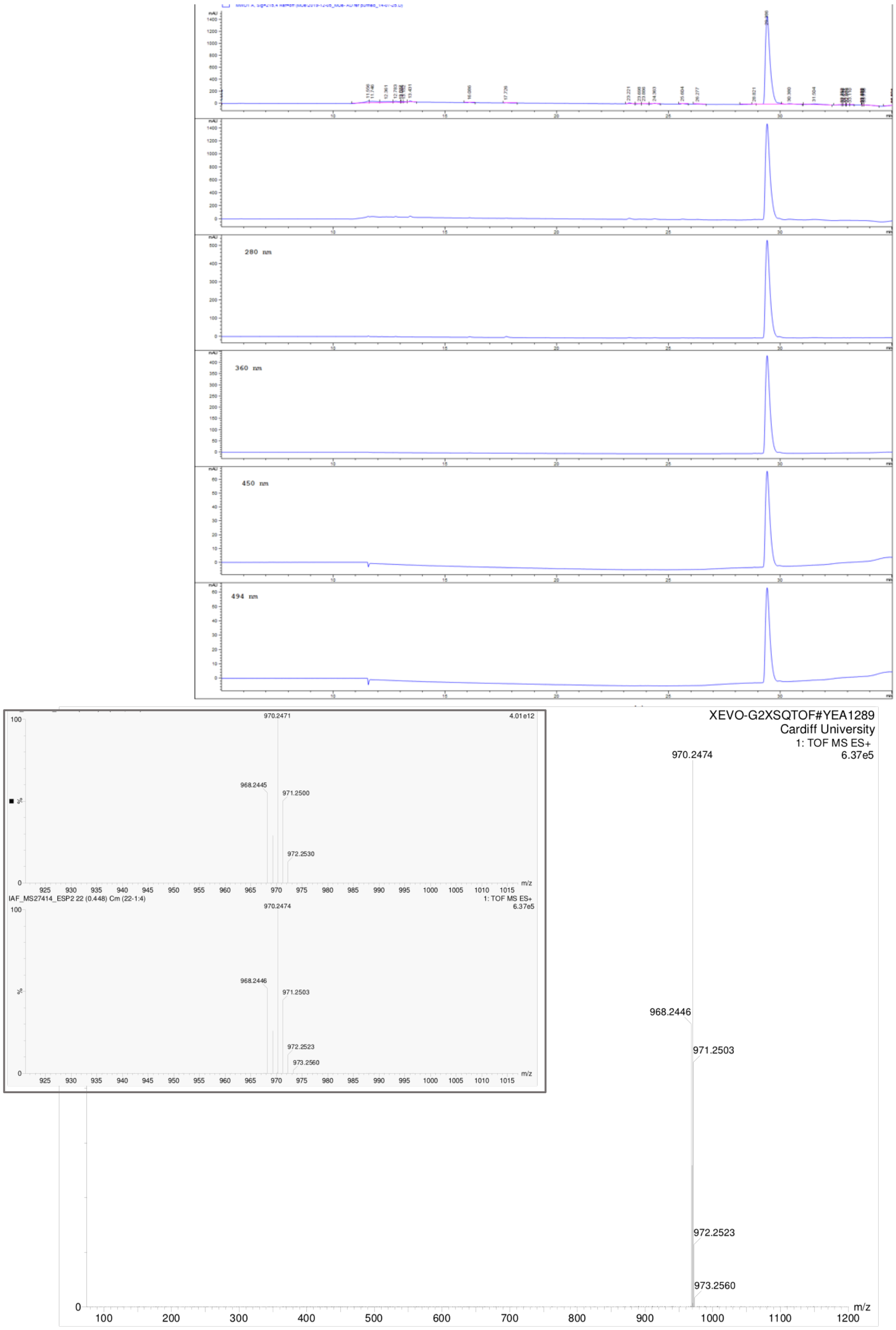

Figure S1. *Top*: HPLC traces of the purified **Ir-PYR** monitored at a series of wavelengths. The gradient is 3.8% MeCN/min (from 100% H_2O to 95% MeCN in 35 min). The column is a C18, 100Å, 5 μm, 10.0 × 250 mm (ACE-121-2510). Product elution was at 29.4 min. *Bottom*: resultant HRMS data (see inset for isotopic match) for the eluted peak corresponding to [$C_{46}H_{35}N_7OF_4{}^{191}Ir$] which requires *m/z* 968.2445.

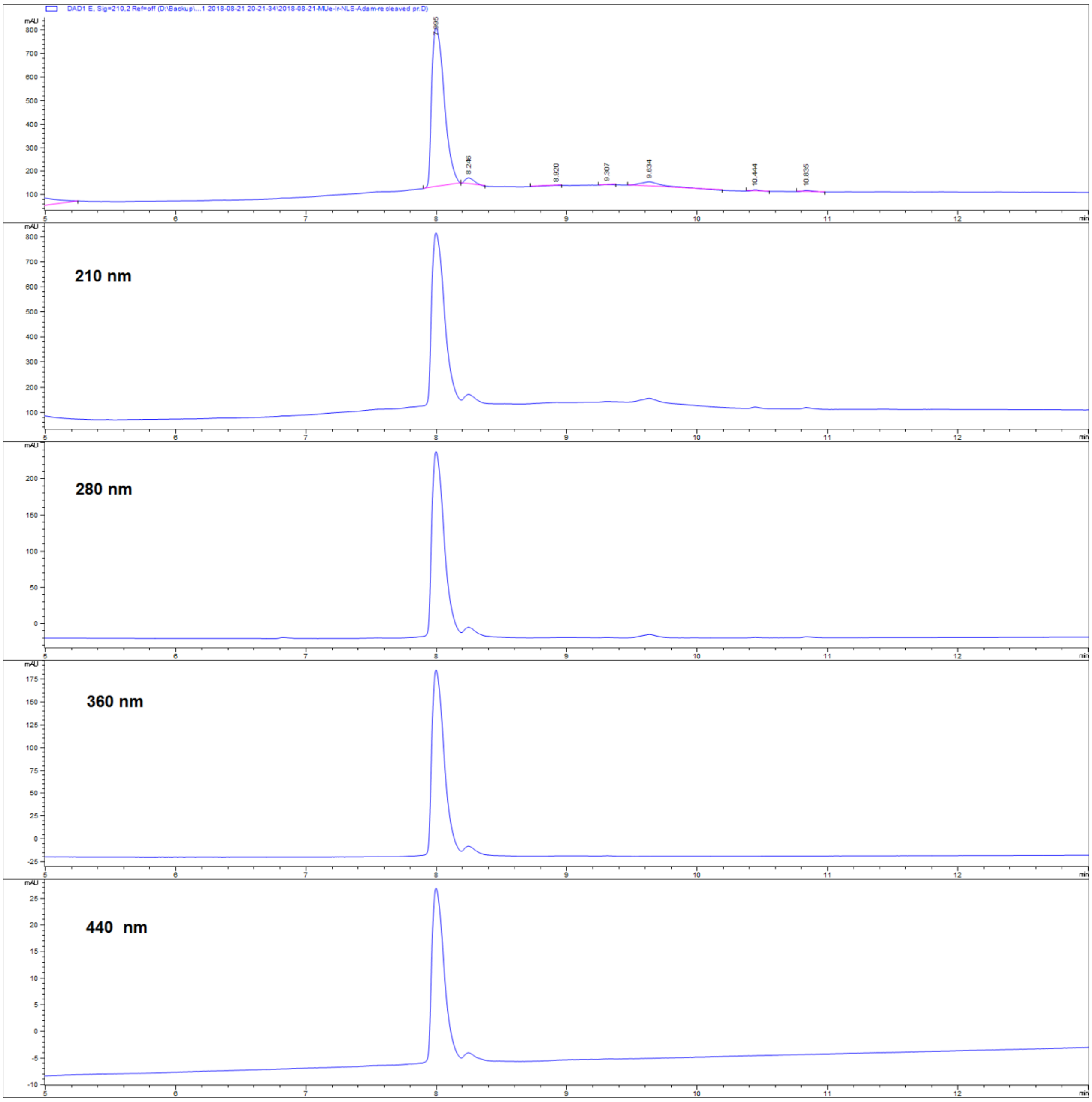

Figure S2. HPLC traces of the purified **PAAKRVKLD** peptide functionalised complex **Ir-CMYC** monitored at a series of wavelengths (440 nm is the signature abosption wavelength for the Ir(III) complex unit). Isolated products were combined and re-cleaved for 1.5 h in 92.5 % TFA, 2.5 % TIS, 2.5 % H_2O, 2.5 % DODT.

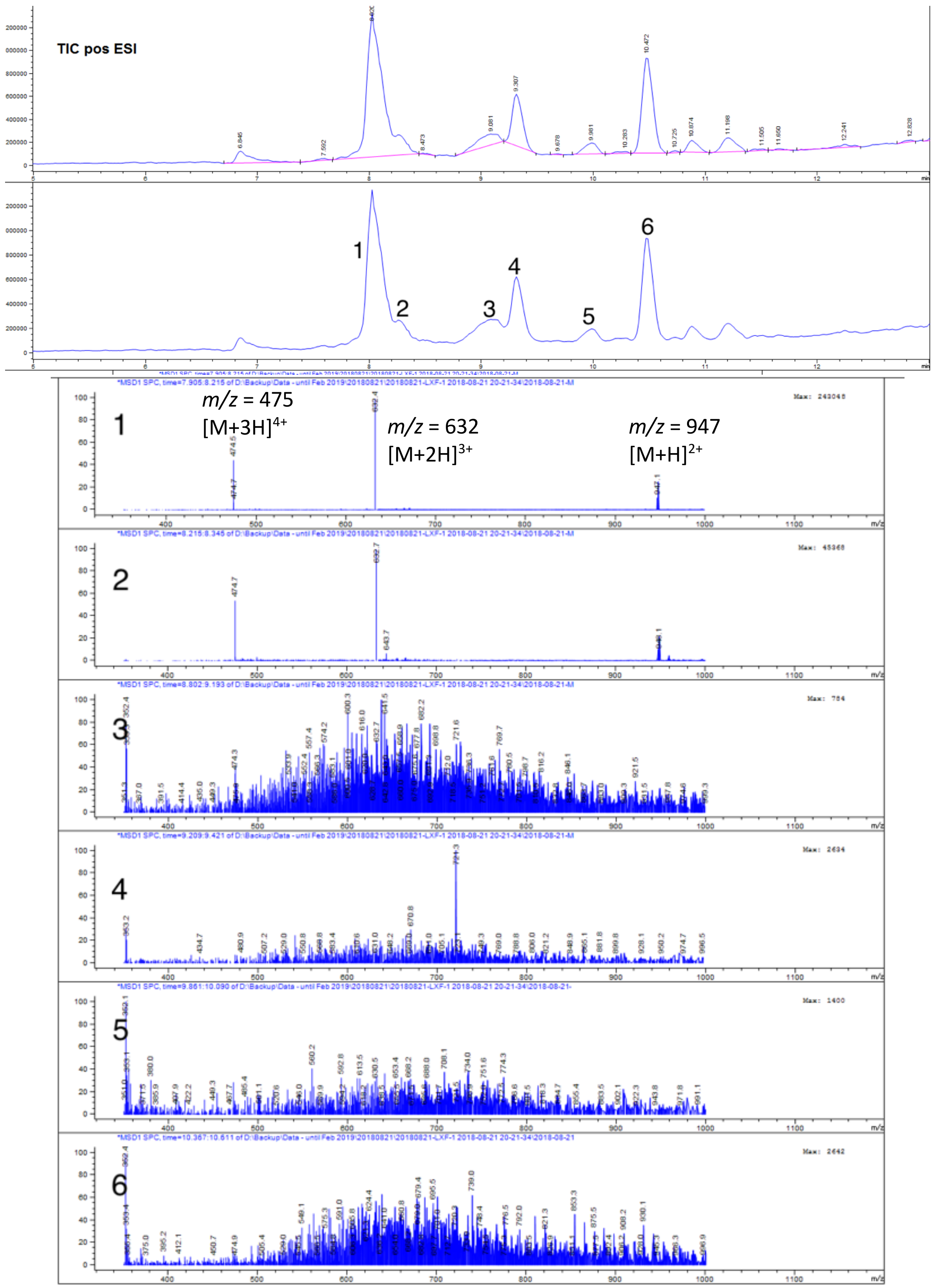

Figure S3. *Top*: Crude HPLC trace (210 nm) for **Ir-CMYC**. *Bottom*: Mass spectra relating to unpurified Ir-CMYC. Peaks **1** and **2** contain Ir-CMYC (rmm = 1895) where protonated species $[M + H]^{2+}$, $[M + 2H]^{3+}$ and $[M + 3H]^{4+}$ were observed at *m/z* = 947, 632 and 475, respectively.

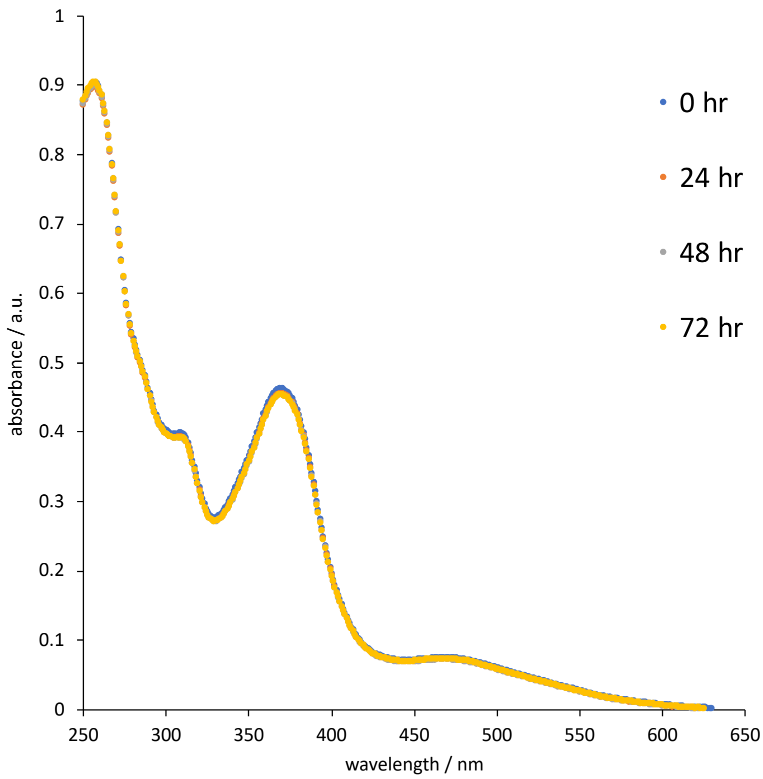

Figure S4. UV-vis. spectra of **Ir-CMYC** in aerated water at 25 °C. The spectra were collected at 0, 24, 48 and 72 hrs and are essentially superimposable.

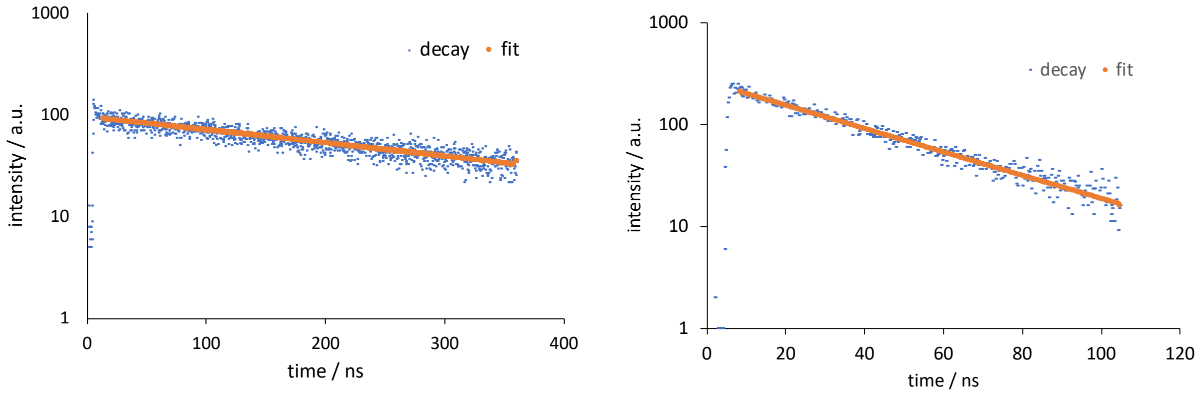

Figure S5. Time resolved luminescence decay profiles (λ_{exc} = 295 nm; λ_{em} = 670 nm). *Left*: **Ir-PYR** in aerated MeCN at 25 °C. The fitted decay gives an observed lifetime of 353 ns. *Right*: **Ir-CMYC** in aerated water at 25 °C. The fitted decay gives an observed lifetime of 38 ns.

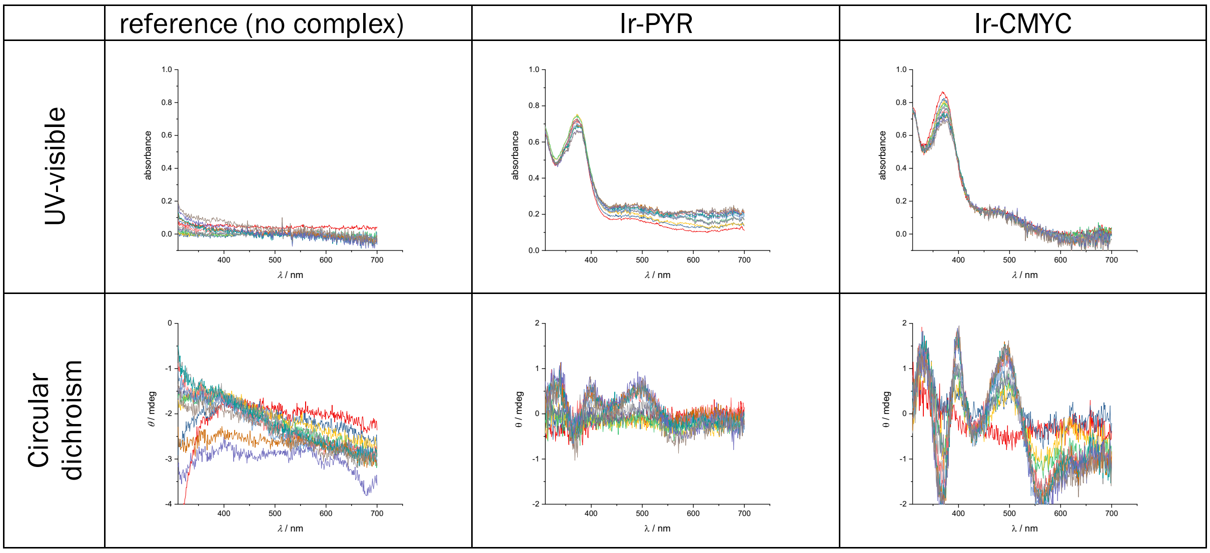

Figure S6. UV-visible and circular dischroism titrations with DNA carried out in buffer (25 mM MOPS, 50 mM NaCl, 1 mM EDTA) at 25 °C.

Tables

Table S1. Fitted binding parameters for **Ir-PYR** and **Ir-CMYC** binding to FS DNA.[a]

	**Ir-PYR**	**Ir-CMYC**
K / 10^3 M^{-1}	1.5 ± 0.7	4.5 ± 1.0
n / base pairs	3 [b]	3 [b]
$\theta_{m,free}$ / mdeg	0 [b]	0 [b]
$\Delta\theta_{m,\ 493\ nm}$ / 10^4 mdeg	3.4 ± 0.7	5.0e4 ± 0.5
$\theta_{background,\ 493\ nm}$ / mdeg	-0.22 ± 0.07	-0.32 ± 0.13
$\Delta\theta_{m,\ x\ nm}$ / mdeg	3.3 ± 0.7[c]	-6.6 ± 0.6[d]
$\theta_{background,\ x\ nm}$ / 10^4 mdeg	-0.28 ± 0.07[c]	0.40 ± 0.14[d]

a) Conditions: in buffer (25 mM MOPS, 50 mM NaCl, 1 mM EDTA) at 25 °C;
b) Parameter restricted to the value given;
c) At 400 nm;
d) At 373 nm.

References

[1] A. D. Edelstein, M. A. Tsuchida, N. Amodaj, H. Pinkard, R. D. Vale, and N. Stuurman, *Journal of Biological Methods,* 2014, **1**, e11.

[2] J. Schindelin, I. Arganda-Carreras, E. Frise, V. Kaynig, M. Longair, T. Pietzsch, S. Preibisch, C. Rueden, S. Saalfeld, B. Schmid, J.-Y. Tinevez, D. J. White, V. Hartenstein, K. Eliceiri, P. Tomancak, A. Cardona, *Nature Methods*, 2019, **9**, 676.

[3] L. A. Mullice, R. H. Laye, L. P. Harding, N. J. Buurma, S. J. A. Pope, *New J. Chem.*, 2008, **32**, 2140

[4] L. Hahn, N.J. Buurma, L.H. Gade, *Chem. Eur. J.*, 2016, **22**, 6314

[5] K.A. Phillips, T.M. Stonelake, K. Chen, Y. Hou, J. Zhao, S. J. Coles, P.N. Horton, S. J. Keane, E.C. Stokes, I. A. Fallis, A. J. Hallett, S.P. O'Kell, J.M. Beames, S.J. A. Pope, *Chem. Eur. J.*, 2018, **24**, 8577.
